# Increase in melanoma knowledge in Latino patients after a targeted digital educational program

**DOI:** 10.1016/j.jdin.2023.11.002

**Published:** 2023-11-30

**Authors:** Adina Greene, Vivian C. Iloabuchi, Elizabeth Stoos, Richard J. Butterfield, Nan Zhang, Aaron R. Mangold, Sancy Leachman, Collin M. Costello

**Affiliations:** a University of Arizona College of Medicine, Phoenix, Arizona; b Department of Dermatology, Mayo Clinic, Scottsdale, Arizona; c Mayo Clinic Alix School of Medicine, Scottsdale, Arizona; d Department of Dermatology and Knight Cancer Institute, Oregon Health & Science University, Portland, Oregon; e Department of Quantitative Health Sciences, Scottsdale, Arizona

**Keywords:** health disparate, melanoma, minority and vulnerable populations, minority groups, minority health

*To the Editor:*

Latinos are the largest US ethnic group and are projected to become one-third of the US population by 2060.^1^ As the US Preventive Services Task Force does not have sufficient evidence to support melanoma screening for the general population, targeted education may serve as a better focus for mitigating skin cancer risk in the Latino population.^2^

The Mayo Clinic Institutional Review Board approved this study. Recruitment included emailing a 4% random sample (4151 patients) of Mayo patients who identified as Latino; additional recruitment included Facebook and Instagram advertisements and posts on the Mayo Clinic’s website and social media channels. The baseline, postintervention, and 3-month follow-up survey questions were adopted from the War on Melanoma, the National Institutes of Health 2015 questionnaire, and Eilers et al.^3^ The material included acral melanomas and Latino patient images and was translated into Spanish. The Melanoma Knowledge Score (MKS) encompassed questions on identifying phenotypes and behaviors associated with melanoma risk, warning signs for melanoma, and how to prevent and detect melanoma early. It was calculated as the sum of correctly answered true items (13) minus incorrectly answered false items (10). A *P* value of <.05 was considered statistically significant.

Patient demographics, baseline sun-protective behaviors, and baseline MKS are presented in Table I for 394 patients who completed the baseline survey. Sunscreen use was reported by 75.3%, with 31.3% reporting daily application. Based on comparison of Fitzpatrick I/II/III (*n* = 175) with Fitzpatrick IV/V/VI (*n* = 206), sunscreen was used by 81.7% and 69.4% of patients, respectively (*P* = .006). In total, 44.6% of patients reported undergoing a skin examination by a dermatologist or other kind of doctor, 19.3% reported being taught to perform a self-skin examination, 11.6% reported performing a head-to-toe self-skin examination in the last 3 months, and 8.5% reported helping a family member or friend with a self-skin examination in the last 3 months.

**Table I:** Entire cohort demographics and baseline sun-protective behaviors (*n* = 394)

Demographics	
Age (y)	
Mean (SD)	60.5 (15.5)
Median (IQR)	62.0 (51-71)
Range	20.0-99.0
Gender, n (%)	
Male	97 (24.6%)
Female	297 (75.4%)
Education level (%)	
High school diploma or some high school without a diploma	10.9%
Some college or technical training	23.6%
Associate or Bachelor’s degree	35.5%
Master’s or Doctorate	30.0%
Fitzpatrick type (%)	
I & II	17.3%
III & IV	49.1%
V & VI	33.6%
Sun-protective behaviors, n (%)	
Do you use sunscreen?	
Yes	292 (75.3%)
No	96 (24.7%)
When do you use sunscreen?	
I use sunscreen daily	90 (31.3%)
I use sunscreen when outside for an extended period of time	198 (68.8%)
Have you ever undergone a skin examination by a dermatologist or other kind of doctor?	
Yes	180 (46.8%)
No	201 (52.2%)
I don’t know	4 (1.0%)
Have you ever been taught to perform a head-to-toe self-skin examination?	
Yes	77 (20.0%)
No	302 (78.4%)
I don’t know	6 (1.6%)
When was your last head-to-toe self-skin examination?	
Within the last month	23 (5.9%)
1 mo to less than 3 mo ago	22 (5.7%)
3 mo to less than 6 mo ago	30 (7.8%)
6 mo to less than 12 mo ago	40 (10.3%)
More than 12 mo ago	77 (19.9%)
Never	195 (50.4%)
Have you helped a loved one perform a self-skin examination in the last 3 months?	
Yes	33(8.5%)
No	353 (91.5%)
How likely are you to use a tanning bed?	
Not likely at all	321 (83.2%)
Not very likely	24 (6.2%)
Somewhat likely	29 (7.5%)
Extremely likely	12 (3.1%)
Melanoma Knowledge Score (points)	
Mean (SD)	8.2 (2.8)
Median (IQR)	9.0 (6.0-10.0)
Range	0.0-13.0

Patient demographics and comparisons between baseline, postintervention, and 3-month follow-up sun-protective behaviors and MKS are presented in Table II. Participants reported an average of 22% increase in their MKS (*P* < .001) and maintained an 11% increase at the 3-month follow-up (*P* < .001).

**Table II:** Three-month follow-up cohort demographics and melanoma knowledge (*n* = 57)

Demographics	
Age (y)	
Mean (SD)	58.3 (13.1)
Median (IQR)	60 (50-68)
Range	27.0-81.0
Gender, n (%)	
Male	12 (21.1%)
Female	45 (78.9%)
Education level (%)	
High school diploma or some high school without a diploma	1.8%
Some college or technical training	22.8%
Associate or Bachelor’s degree	28.1%
Master’s or Doctorate	45.6%
Melanoma Knowledge Score (points)	
Mean (SD)	10.1 (2.1)
Median (IQR)	10 (9.0-11.0)
Range	4.0-13.0

Our study demonstrates that developing melanoma educational materials that are culturally relevant and focused on Latino populations can provide persistent improvements in melanoma knowledge, sun-protective behaviors, self-skin examinations, and helping friends or family members perform self-skin examinations. However, MKS and sun-protective behaviors decreased at 3 months, suggesting that repeated education or reminders are needed to retain long-term, durable responses. To our knowledge, this is the first Latino melanoma educational program leveraging social media. Latinos at higher risk of sun-induced melanoma will likely benefit from self-full-body self-skin examination and daily sunscreen use; however, Latinos at low risk of sun-induced melanoma may derive the most benefit from a focused acral and mucosal self-examination.^4^^,^^5^ The decrease in retention rates between baseline and postintervention questionnaires was a limitation.

Our study demonstrates that developing melanoma educational materials that are culturally relevant and focused on Latino populations can provide persistent improvements in melanoma knowledge.

## Conflicts of interest

Author Costello has no relevant disclosures; unrelated to this study, he is an investigator for DeepX Health. Author Mangold has no relevant disclosures; he has consulted for Kyowa, Eli Lilly, Momenta, UCB, and Regeneron in the past, greater than 24 months ago; he has consulted for PHELEC in the past, greater than 12 months; he has consulted for Incyte, Soligenix, Clarivate, and Bristol Myers Squibb in the past, less than 12 months ago; he consults for Argenyx, Boehringer, Janssen, and Ingelheim currently; he consults for Regeneron and Pfizer currently with payments to the institution; he has grant support from Kyowa, Miragen, Regeneron, Corbus, Pfizer, Incyte, Eli Lilly, Aregenx, Palbella, Abbvie, Priovant, Merck in the last 24 months; beyond 24 months, grant support has come from Sun Pharma, Elorac, Novartis, and Janssen; and his current patents include Methods and Materials for assessing and treating cutaneous squamous cell carcinoma (provisional 63-423254), use of oral JAKi in Lichen Planus (provisional 63/453,065), and Topical Ruxolitinib in Lichen Planus (WO2022072814A1). Author Leachman has received unrestricted educational grant 18 months ago from Caste Biosciences and has been compensated for Orlucent MSAB within 24 months, and Merk MSAB co within 18 months; she has also served as the Co-I on Veriskin: clinical protocol uncompensated, Palvella: MSAB, Local CO-I for Pachyonychia congenita therapeutic uncompensated, and currently is working on Pathology Watch: MSAB research project support uncompensated. All other authors have no conflicts of interest and no financial disclosures.
